# Intima-Media Thickness in the Carotid and Femoral Arteries for Detection of Arteriosclerosis in Human Immunodeficiency Virus-Positive Individuals

**DOI:** 10.5935/abc.20160197

**Published:** 2017-01

**Authors:** Emmanuelle Tenório Albuquerque Madruga Godoi, Carlos Teixeira Brandt, Heloisa Ramos Lacerda, Jocelene Tenório Albuquerque Madruga Godoi, Dinaldo Cavalcanti de Oliveira, Gabriela Farias Araujo Sousa Costa, Gerson Gomes dos Santos Junior, Kaliene Maria Estevão Leite, Juannicelle Tenório Albuquerque Madruga Godoi, Adriana Ferraz de Vasconcelos

**Affiliations:** Universidade Federal de Pernambuco (UFPE), Recife, PE - Brazil

**Keywords:** Carotid Artery Disease, Atherosclerosis, Carotid Intima-Media Thickness, HIV, Ankle Brachial Index

## Abstract

**Background:**

The prevalence of atherosclerosis is higher in HIV-positive people, who also experience it earlier than the general population.

**Objectives:**

To assess and compare the prevalence of atherosclerosis evaluated by the intima-media thickness of carotid and femoral arteries, and by the ankle-brachial pressure index (ABPI) in HIV patients treated or not treated with protease inhibitors (PIs) and controls.

**Methods:**

Eighty HIV+ subjects (40 using PIs and 40 not using PIs) and 65 controls were included in the study. Atherosclerosis was diagnosed by (carotid and femoral) ITM measurement and ABPI. Classical risk factors for atherosclerosis and HIV were compared between the groups by statistical tests. A p ≤ 0.05 was considered significant.

**Results:**

An IMT > P_75_ or the presence of plaque was higher in the HIV+ than in the control group (37.5% vs 19%, p = 0.04). Comparative analysis showed a significant difference (p=0.014) in carotid IMT between HIV+ with PIs (0.71 ± 0.28 mm), without PIs 0.63 ± 0.11 mm and, and controls (0.59 ± 0.11 mm). There was no significant difference in femoral IMT between the groups or in ABPI between HIV+ subjects and controls. However, a significant difference (p=0.015) was found between HIV+ patients not treated with PIs (1.17 [1.08 - 1.23]), and controls 1.08 [1.07 - 1.17]).

**Conclusion:**

In HIV patients, atherosclerosis is more prevalent and seems to occur earlier with particular characteristics compared with HIV-negative subjects.

## Introduction

HIV-positive individuals (HIV+) experience different conditions in terms of morbidity and mortality of atherosclerosis and related cardiovascular events, as compared with subjects free of infection.^1,2^ Cardiovascular disease (CVD), particularly atherosclerotic disease, is more prevalent and occurs earlier in HIV+ individuals than in those without the infection.^3-5^

The traditional risk factors for CVD are age, male sex, smoking, diabetes mellitus (DM), dyslipidemia, and systemic arterial hypertension (SAH). Studies have shown that these factors may be more prevalent in HIV+ people.^6,7^

The highly active antiretroviral therapy (HAART) is associated with a variety of adverse effects, which have proatherogenic effects and are also associated with CVD.^8,9^ While some authors have suggested that protease inhibitors (PIs) may be associated with early atherosclerosis and CVD,^10^ others have shown that the lipid profile is less affected by newer medications, which hence mitigates the increase in cardiovascular risk by endothelial dysfunction.^11^ In a study comparing HIV+ subjects with dyslipidemia treated with PIs with healthy controls, no difference was found in endothelial function between the groups.^12^

Intima-media thickness (IMT) is a non-invasive, early marker of atherosclerosis; an increase in this measure may reflect an increase in cardiovascular risk.^13^ This is an independent predictor of CVD, and may be considered as a marker for the assessment of subclinical atherosclerosis, including in HIV+ individuals.^14^ In addition, common femoral and right subclavian arteries have been used for IMT measurements, and suggested as early markers of atherosclerosis.^15-17^

The ankle-brachial pressure index (ABPI) is a simple, non-invasive method, with high predictive value of peripheral artery disease (PAD) and cardiac disease. Values lower than 0.9 are associated with a significant increase in cardiovascular risk, independent of other risk factors.^18^

The detection of subclinical atherosclerosis allows a more adequate approach of HIV+ patients at risk for cardiovascular disease.

In the present study, the primary objective was to assess the prevalence of atherosclerosis by IMT of the common carotid and femoral arteries, and by the ABPI. The secondary objective was to compare classical risk factors of atherosclerosis and HIV-specific risk factors between patients treated and not treated with PIs.

## Methods

### Study design and population

This is a cross-sectional, prospective, analytical study on HIV+ patients in HAART including or not PIs during the period from June 2015 to February 2016. The sample was empirically determined by the authors based on the literature on the theme,^19,20^ and included 40 HIV+ patients in HAART with PIs, 40 HIV+ patients in HAART without PIs, and 65 controls.

### Phases of the study

Patients were selected at the outpatient service of infectious and parasitic diseases of this institution according to inclusion and exclusion criteria. For reference of outcome measures, we included 65 healthy subjects, who were sex- and age-matched with HIV+ patients - 40 patients in use of PIs were matched with 40 controls, whereas 40 patients not using PIs were matched with 25 controls. The HIV+ patients were enrolled in the program of prevention, control and treatment of AIDS, and control group was composed of individuals accompanying patients.

Inclusion criterion of HIV+ patients was time on HAART of five years or more, and the exclusion criteria were history of cardiovascular diseases - angina pectoris, acute myocardial infarction (AMI), stroke, PAD, hospitalization in the last two months, very low CD4 levels and/or very high viral load (VL). Exclusion criteria of healthy volunteers were history of cardiovascular diseases, smoking, DM and/or SAH.

Clinical data and complementary tests were collected by questionnaires, and detailed data about the treatment were obtained from patients' medical records. Participants had a Framingham risk score lower than 10% (low risk).

The atherosclerosis risk factors assessed were SAH, smoking, DM, hypercholesterolemia, hypertriglyceridemia, and history of any cardiovascular event - AMI, angina, stroke or PAD. Obesity was assessed by body mass index (BMI). A BMI between 18.5 and 24.9 kg/m^2^ was considered healthy weight, and a BMI between 25.0 and 29.9 kg/m^2^ was considered overweight. HIV-related factors assessed were current CD4, current VL, time of disease, time of treatment and type of HAART.

The study was approved by the local Ethics Committee, and all participants signed the informed consent form.

### Protocol of intima-media thickness measurement

Ultrasonographic assessment of common carotid IMT was performed by B-mode ultrasonography (LOGIQ*e*, DICOM with a 12-RS linear transducer, General Electric^®^), by a blinded observer. The common carotid IMT measurement was used as reference. Common carotid was analyzed by cross sections and longitudinal sections from the proximal segment of the common carotid to the bifurcation of the internal and external carotid arteries. The IMT measurement was performed in the posterior wall of the common carotid, in an area free of plaque, defined as the distance between two echogenic lines represented by the lumen-intima interface and media-adventitia interface of the arterial wall. The mean value and the maximum value are commonly used as references for IMT measured in the common and internal carotid, respectively.^15^ Carotid atherosclerotic plaque was defined as a focal structure that extends at least 0.5 mm into the arterial lumen and/or measures 50% or more of the adjacent IMT value and/or has an IMT value greater than 1.5 mm.^15^ The automated measurement of IMT was determined, using a software, in the right and left common carotid arteries in mean, medium and minimum value. When atheromatous plaques were identified, IMT was determined both automatically and manually. The mean automated measurement of the thickest common carotid artery was used as reference, be it the right or the left artery. As our study population was composed of patients aged less than 65 years, thickened intima-media was defined as an IMT equal to or higher than 0.8 mm.^17,21,22^ The 75 percentile of the study group was also calculated,^15^ and the presence of atheromatous plaque was defined as an IMT higher than 1.5 mm.^15^ The IMT was also measured in the right common femoral artery (RCFA) and left common femoral artery (LCFA), using the same criteria for IMT and atheromatous plaque definition.

### Protocol of measurement of the ankle-brachial pressure index

ABPI was calculated after measurement of right and left ankle pressure, which was measured at the dorsalis pedis artery and the posterior tibial artery. Pressures in the upper and lower extremities were measured using a sphygmomanometer (Becton Dickinson^®^) and a Doppler ultrasound device as above described. The ABPI was calculated by dividing the highest systolic ankle pressure by the highest systolic pressure of the arms. An ABPI of 0.9-1.3 was considered normal; ABPI values above 1.3 indicated incompressible arteries, and values below 0.9 were an indication of PAD.^21^

### Statistical analysis

The Kolmogorov-Smirnov test was used to test the normality of data. Normally distributed data were presented as mean and standard deviation, and data without a normal distribution were expressed as median and minimum and maximum values. A descriptive and analytical analysis of the data was performed. A p ≤ 0.05 was considered significant.

The prevalence and 95% confidence interval of atherosclerosis were calculated in the group of HIV+ patients and controls, and the compared by the Pearson's chi-square test.

The odds ratios (ORs) were calculated taking the control group as reference, and the OR of each HIV+ group (with PIs and without PIs) was estimated.

In between-groups comparison of classical risk factors for atherosclerosis, age and BMI were compared by ANOVA and Bonferroni's post-test. Median values were compared by the nonparametric Kruskal Wallis test, and categorical variables by Pearson's chi-square test.

The chance of atherosclerosis according to HIV and use of PIs was assessed by multivariate logistic regression analysis, adjusted by skin color, hypercholesterolemia, hypertriglyceridemia, DM and BMI. These variables were significantly different between the groups.

For IMT validation analysis, the common carotid IMT was used as reference, and the ROC curve was applied to determine the femoral IMT cutoff. The analysis was performed by c-statistic (area under the ROC curve), measurement of sensitivity, specificity, positive predictive value (PPV) and negative predictive value (NPV), using the using the Stata software, version 12.0.

## Results

Forty subjects with HIV/AIDS in HAART with PIs (26 men, mean age of 42.7 ± 8.8 years), 40 subjects with HIV/AIDS in HAART without PIs (21 men, mean age of 42.2 ± 9.1 years), and 65 controls (37 men, mean age of 39.7 ± 9.7 years) were recruited.

The 75 percentile calculated for the total sample was 0.66. When the IMT was increased (> 0.66 mm), comparative analysis of the IMT of carotid arteries between control, HIV+ with PIs and HIV+ without PIs groups revealed an IMT of 0.59 ± 0.11 mm *vs*. 0.63 ± 0.11 mm *vs*. 0.71 ± 0.28 mm, respectively (p=0.014) (Table 1). The presence of IMT > P75 or plaque was detected in 19.0% (9.1-29.0) in controls, and in 37.5% (21.8 - 53.2) of HIV+ without PIs patients (p = 0.041) (Table 1).

**Table 1:** Prevalence of atherosclerosis assessed by common carotid and femoral intima-media thickness (IMT) and ankle-brachial pressure index (ABPI) in HIV-negative subjects, and HIV-positive subjects in antiretroviral therapy treated or not treated with protease inhibitor (PI)

Atherosclerosis							OR (95%CI)^b^ p value (control *x* without PI)	OR (95%CI)^b^ valor de p (control *x* with PI)	p value (3 groups)
	HIV-negative subjects % (95%CI)	HIV-positive subjects % (95%CI)	HIV carriers			OR control *x* HIV (95%CI)^b^ P value			
			Without PI % (95%CI)		With PI % (95%CI)				
**Intima-media thickness**									
**Carotid**									
IMT	0.59 ± 0.11	0.70 ± 0.27	0.63 ± 0.11		0.71 ± 0.28	0.004	0.007	0.006	0.014
IMT >75P or presence of plaque	19.0% (9.1 - 29.0)	35.0% (24.3 - 45.7)	37.5% (21.8 - 53.2)		32.5% (17.3 47.7)	2.28 (1.05 - 4.98) p = 0.037	2.55 (1.04 - 6.25) p = 0.041	2.05 (0.82 -6.25) p = 0.124	0.095
IMT >0.8mm or presence of plaque	3.2% (0.0 - 7.6)	15.0% (7.0 - 23.0)	15.0% (3.4 - 26.6)		15.0% (3.4 - 26.6)	5.38 (1.16 - 25.1) p = 0.032	5.38 (1.03 - 28.1) p = 0.046	5.38 (1.03 - 28.1) p = 0.046	0.061
**Femoral**									
IMT^d^	0.74 ± 0.30	0.79 ± 0.33	0.75 ± 0.27		0.82 ± 0.38	0.373	0.661	0.155	0.351
IMT >75P or presence of plaque	50.8% (38.1 -63.5)	65.0% (54.3 - 75.7)	60.0% (44.1 - 75.9)		70.0% (55.1 - 84.8)	1.80 (0.92 - 3.53) p = 0.088	1.45 (0.65 -3.24) p = 0.361	2.26 (0.98 -5.22) p = 0.056	0.153
IMT >0.8mm or presence of plaque	11.1% (3.1 - 19.1)	20.0% (11.0 - 29.0)	20.0% (7.0 32.9)		20.0% (7.0 - 32.9)	2.00 (0.77 - 5.21) p = 0.156	2.00 (0.66 - 6.03) p = 0.218	2.00 (0.66 - 6.03) p = 0.218	0.356
**Changes in ABPI^c^**									
ABPI^e^	1.08 (1.07; 1.17)	1.15 (1.08; 1.2)	1.17 (1.08; 1.23)		1.08 (1.07; 1.17)	0.190	0.015	0.797	0.019^a^
Normal (0.9 – 1.3)	95.2% (92.0 - 100)	96.3% (92.0 - 100)	90.0% (80.3 - 99.9)		100% (-)	Reference	Reference	Reference	-
Incompressible (> 1,3)	4.8% (0.0 - 10.2)	5.0% (0.1 - 9.9)	10.0% (0.03 - 19.7)		0% (-)	1.05 (0.23 - 4.88) p = 0.948	2.22 (0.47 - 10.5) p = 0.314	Not calculated	0.116

ABPI: ankle-brachial blood pressure index; OR: Odds Ratios; HIV: human immunodeficiency virus.

a 75 Percentile of IMT of 0.66 mm in the study population;

b Reference group: HIV-negative subjects;

c IMT < 0.9 was not detected in the study group;

d Data with logarithmic transformation for normalization of distribution;

e Median (P25; P75) - Kruskal-Wallis test for between-group comparisons.

The prevalence of IMT > 0.8 mm or atherosclerotic plaque in carotid arteries was 3.2% (0.0-7.6) in the control group and 15% (7.0-23.0) in HIV+, p=0.032. Between-groups comparisons of some of the IMT in carotid and femoral arteries and ABPI data are presented in Table 1.

No significant difference in ABPI was found between the control and HIV+ groups. The ABPI in the control group and HIV+ without PIs group was 1.08 (1.07 - 1.17) *vs* 1.17 (1.08 - 1.23), respectively (p = 0.015). The IMT in femoral artery was 0.74 mm ± 0.30 *vs*. 0.79 mm ± 0.33 in the control and HIV+ with PIs, respectively (p = 0.373) (Table 1).

The following risk factors for atherosclerosis were identified in the 80 HIV+ patients: smoking in 6 patients (7.5%), SAH in 7 (8.75%), hypercholesterolemia in 15 (18.75%), hypertriglyceridemia in 24 (30%) and DM in 6 (7.5%). Mean BMI was within the normal range in the HIV+ with PIs, at the lower limit for overweight in the group without PIs and classified as overweight in the control group. The BMI in the control, HIV+ without and with PIs was 26.2 ± 5.4 *vs*. 25 ± 3.6 *vs*. 24.7 ± 3.7 kg/m^2^, respectively (p = 0.193) (Table 2).

**Table 2:** - Comparison of classical risk factors for atherosclerosis between HIV-negative subjects and HIV-positive subjects in antiretroviral therapy treated or not treated with protease inhibitor (PI)

Atherosclerosis risk factors	Groups			p value (control *x* without PI)	p value (control *x* with PI)	p value (3 groups)
	HIV-negative subjects (n = 63)	HIV-positive subjects				
		Without PI (n = 40)	With PI (n = 40)			
Age, years (mean ± SD)^b^	39.7 ± 9.7	42.2 ± 9.1	42.7 ± 8.8	0.550	0.351	0.215
Male sex (%)	37 (58.7%)	21 (52.5%)	26 (65.0%)	0.998	0.276	0.525
**Years of school (%)**						
0 - 4 years	55 (87.3%)	33 (82.5%)	33 (82.5%)	0.501	0.501	0.732
5 - 7 years	8 (12.7%)	7 (17.5%)	7 (17.5%)			
**Skin color (%)**						
White	27 (42.9%)	8 (20.0%)	10 (25.0%)	0.017^a^	0.066	0.030^a^
Not white	36 (57.1%)	32 (80.0%)	30 (75.0%)			
Smoking (%)	0 (0%)	4 (10.0%)	2 (5.0%)	Not calculated	Not calculated	0.046^a^
SAH (%)	0 (0%)	2 (5.0%)	5 (12.5%)	Not calculated	Not calculated	0.062
Hypercholesterolemia (%)	3 (4.8%)	3 (7.5%)	12 (30.0%)	0.563	<0.001^a^	<0.001^a^
Hypertriglyceridemia (%)	1 (1.6%)	2 (5.0%)	22 (55.0%)	0.315	<0.001^a^	<0.001^a^
Diabetes (%)	0 (0%)	1 (2.5%)	5 (12.5%)	Not calculated	Not calculated	0.007^a^
BMI, kg/m^2^ (mean ± SD)^b^	26.2 ± 5.4	25.0 ± 3.6	24.7 ± 3.7	0.585	0.285	0.193
Overweight/obese (%)	36 (57.1%)	19 (47.5%)	19 (47.5%)	0.339	0.339	0.519

a ANOVA with Bonferroni post-test;

b Despite the significant difference between the groups, the analyses were not adjusted for hypercholesterolemia, hypertriglyceridemia and race conditions, due to the low frequency  of the variables. HIV: human immunodeficiency virus; HAS: systemic arterial hypertension; BMI: body mass index; SD: standard deviation.

The time of disease was significantly different between the HIV+ with PIs (13.6 ± 6.2 years) as compared with the HIV + without PIs group (7.3 ± 6.8 years) (p<0.001). The time of HAART was 12.1 ± 6.7 years in the group with PIs *vs*. 6.6 ± 6.7 years in the group without PIs, p < 0.001. Only 4 (5%) HIV+ patients had CD4 levels lower than 200, and only 7 (8.75%) of studied population had a detectable VL sample, with a maximum of 3,231 copies (Table 3).

**Table 3:** Comparison of risk factors, laboratory parameters, and HIV-related data between HIV-positive subjects in antiretroviral therapy treated with protease inhibitor and not treated with protease inhibitor (PI)

Factors	Without PI	With PI	p value
**Laboratory^a^**			
Cholesterol (mg/dL)^b^	182.3 ± 32.9	188.0 ± 57.9	0.642
HDL cholesterol (mg/dl)^b^	52.5 ± 13.6	45.4 ± 10.6	0.071
LDL cholesterol (mg/dl)^b^	103.2 ± 31.0	81.8 ± 25.2	0.044
Triglycerides (mg/dL)^c^	95.7 (73.6; 143.5)	238.1 (140; 375.9)	<0.001
**Related to HIV**			
Time of HIV infection (in years)^b^	7.27 ± 6.78	13.62 ± 6.20	<0.001
Time of HAART (in years)^b^	6.62 ± 6.65	12.1 ± 6.69	<0.001
**CD4 count (%)**			
< 200 cells/mm^3^	2 (5.0%)	2 (5.0%)	0.599
200 - 500 cells/mm^3^	9 (22.5%)	13 (32.5%)	
> 500 cells/mm^3^	29 (72.5%)	25 (62.5%)	

a Thirty-six of 40 patients in the group without PI, and 20 of 40 patients without PI had available laboratory data;

b Mean ± standard deviation; Student's t-test;

c Median (P25; P75) - nonparametric Mann-Whitney test. HIV: human immunodeficiency virus; HDL: *High Density Lipoprotein; *LDL: * Low Density Lipoprotein;* HAART: highly active antiretroviral therapy.

**Figure 1:**
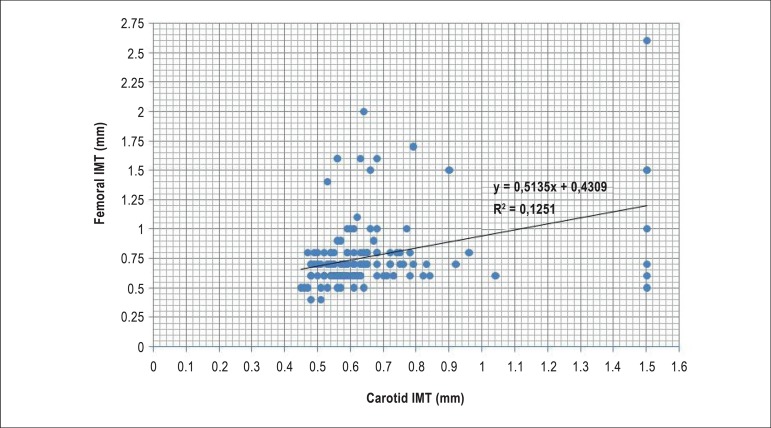
Correlation between intima-media thickness (IMT) in the common carotid and in the common femoral artery; Pearson correlation = 0.354 (p < 0.001)

There was a positive Pearson correlation between common carotid IMT and femoral carotid IMT ([ρ = 0.354 (p<0.001)] (Figure 1).

**Figure 2:**
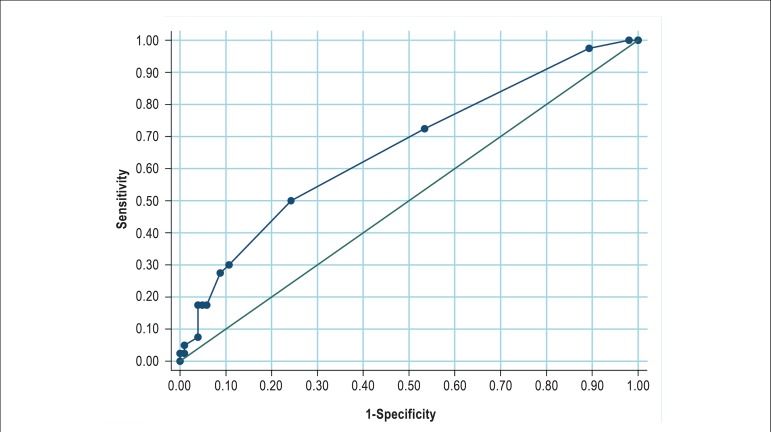
ROC curve of femoral artery intima-media thickness (IMT), considering 'atherosclerosis' as an IMT in the common carotid above 0.66 mm; *Area under the curve = 0.6614 (95%CI: 0.563 - 0.760)

Figure 2 shows the ROC curve of IMT in femoral artery, considering atherosclerosis as the carotid artery IMT >0.66 mm. Using a cutoff of 0.7mm in the femoral artery, we observed a 72.5% sensitivity, 46.6% specificity, area under the ROC curve of 0.661 and kappa of 14.3% (Table 4).

**Table 4:** Accuracy of intima-media thickness (IMT) in the femoral and carotid arteries in the studied patients

	Carotid IMT^a^		Statistics (95%CI)
	With atherosclerosis	Without atherosclerosis	
Femoral IMT^b^			
With atherosclerosis	29	55	Sensitivity: 72.5% (58.0 - 86.9)
Without atherosclerosis	11	48	Specificity: 46.6% (36.8 - 56.4)
Total	40	103	PPV: 34.5% (24.1 - 44.9)
			VPN: 18.6% (8.4 - 28.9)
			Area under the curve: 0.661 (0.563 - 0.760)
			Kappa: 14.3% (0.8 - 27.8)

a Atherosclerosis by the 75 percentile of IMT in the study population equal to or greater than 0.66 mm;

b Atherosclerosis by the femoral IMT cutoff equal to or greater than 0.7 mm ,estimated by the ROC curve. PPV: positive predictive value; NPV: negative predictive value.

## Discussion

The present study demonstrated that the IMT of carotid arteries was greater in HIV+ patients as compared with controls, regardless of the use of PIs.

Eira et al.^20^ studied 118 patients divided into 4 groups (HIV patients in HAART, HIV patients without treatment - naïve group, noninsulin-dependent diabetes and control). Right carotid IMT was greater in the naïve group than in HAART and control groups (0.55 ± 0.02 mm *vs*. 0.52 ± 0.02 mm *vs* 0.52 ± 0.02 mm, respectively; p<0.0011), whereas the IMT in the left carotid artery was greater in HAART group than in naïve and DM groups (0.64 ± 0.04 mm *vs*. 0.53 ± 0.04 mm *vs*. 0.52 ± 0.04 mm, respectively; p<0.0001). Therefore, these results are in agreement with ours by showing greater IMT in HIV+ patients than in other groups.^20^ Other authors have also reported higher prevalence of atherosclerosis in HIV+ patients treated with HAART.^23^

Our findings differ from those of Godoi et al.^21,22^ on 70 HIV patients and 70 controls, showing no difference between these groups. However, the study involved younger subjects, and included smoking, hypertensive and DM controls, which may have contributed to greater IMT values.

In our study, HIV+ patients had a mean IMT higher than the 75 percentile of the study group and the control group.

The 75 percentile depends on the studied population, as it varies with sex, race and age. In the Elsa-Brasil study, this parameter was estimated in pardo, male subjects with similar age as our study group; the 75 percentile was 0.58 - 0.63 mm.^15^

In HIV+ patients, chronic immune activation and chronic inflammation are associated with increased risk for atherosclerosis. Ultrasonography was one of the first diagnostic methods to identify high incidence of subclinical atherosclerosis in HIV-infected individuals as compared with healthy controls.^24,25^

It has been hypothesized that the HAART activates endothelial function and promotes atherosclerosis. Thus, HIV, immune reconstitution response and HAART may promote early endothelial activation, and hence represent proatherogenic factors and/or accelerators of atherosclerosis.^26,27^ In our study, there was no significant difference in IMT between the HIV+ patients treated with PIs and not treated with PIs. Despite the hypothesis of HAART-related endothelial dysfunction, many issues need further clarification.

Nolani et al.^12^ compared HIV-infected subjects with dyslipidemia treated with PIs and healthy controls and did not find any difference between the groups.

Although our HIV+ patients using PIs had a significantly longer time of treatment compared with the HIV+ patients without IPs, apparently, the use of these medications did not affect the IMT in HIV+ patients.

In some situations, the treatment of HIV+ patients may be started without PIs. In the present study, the possibility that patients treated with PIs had been previously treated without these drugs cannot be ruled out, which may explain the longer time of disease and treatment in this group.

Newer PIs are relatively less related with metabolic disturbances, and hence, have a smaller effect on the increase in cardiovascular risk induced by endothelial dysfunction.^28,29^ This may explain the absence of a significant difference in IMT between the patients treated and not treated with PIs in the present study.

The principles of the HAART are viral control and stabilization of the immune system, resulting in increased life expectancy and reduction of opportunistic infections.^1^ HIV patients have cardiovascular changes caused by exposure to classical CVD risk factors, virus infection and cardiotoxicity to antiretroviral drugs.^8^ The HIV infected patients in our study were immunologically stable. Most of them had CD4 levels above 200 cells/ mm^3^, and only seven patients had a detectable VL. There was no significant difference in CD4 and VL between patients with PIs and without PIs.

Previous studies^3-5^ have demonstrated that atherosclerosis is more prevalent and occurs earlier in patients with HIV infection as compared with non-infected individuals, which are in agreement with our findings.

The prevalence of hypercholesterolemia and hypertriglyceridemia was higher in HIV+ patients using PIs than in other groups, suggesting that the use of PIs may affect these parameters.

The high prevalence of ABPI > 1.3 in HIV+ subjects may be influenced and mediated by vascular elasticity and atheroma plaque formation.^10^ In the present study, no significant difference in ABPI was detected between HIV groups and controls, which may be explained by impairment of vessel wall elasticity. The ABPI in HIV+ patients without PIs was within normal range.

A metanalysis investigated the ABPI in HIV-infected patients. Variable selection criteria of the study groups were used in the studies, and there was no consensus on the risk factors for an abnormal ABPI. The prevalence of increased ABPI was higher in HIV+ patients than in the general population. In addition, it has not been established whether a high prevalence of altered ABPI is associated with high incidence of cardiovascular events.^30^

The common carotid IMT is still the reference for other artery measures, and has been shown the best accuracy in studies correlating increased cardiovascular risk and HIV infection. The IMT of common femoral artery and right subclavian origin may also be considered and suggested by some authors as an early marker of atheromatosis. However, these parameters do not combine both high specificity and high sensitivity, which was corroborated by our study.^16,17,22^

When the IMT in the common carotid artery and femoral arteries were compared, we found a positive but weak correlation between the groups. We believe that further studies involving a higher number of patients would improve the understanding of this correlation. Another study^17^ has demonstrated a correlation (Pearson correlation), also not strong, between common carotid artery and right subclavian artery.

In this study, the ROC curve was used to compare two tests, the common carotid IMT (used as reference) and the femoral artery IMT. This analysis allows the comparison of two or more diagnostic tests, which is one of the greatest advantages of the method.^31^

By using a cutoff > 0.7mm for IMT, we tested the accuracy of IMT in the femoral and carotid arteries in HIV+ patients. Based on sensitivity, specificity, PPV and NPV, the IMT in the femoral artery could not be used as a surrogate for the measurement in the carotid artery.

The main limitations of this study were the empirical definition of the population sample size, and the study design that does not allow the establishment of a cause-effect relationship. It is worth mentioning, however, that other studies on the theme, available in the literature, have been performed on smaller sample sizes. In addition, whether the fact that we did not evaluate pulse wave velocity or flow mediated dilation of the brachial artery, which are also non-invasive methods for the diagnosis of subclinical atherosclerosis, may represent a limitation of the study, is a matter of discussion. Nevertheless, there are reliable data on the role of IMT as a cardiovascular risk predictor.^19,20^

## Conclusions

Higher values of IMT and higher prevalence of IMT above the 75 percentile and IMT > 0.8 mm or presence of atherosclerotic plaque in HIV+ patients suggest an earlier occurrence of atherosclerosis in this population as compared with healthy controls. However, no difference was found in the occurrence of abnormal ABPI between the groups.

The prevalence of smoking was higher in HIV+ patients without PIs, whereas cholesterolemia, hypertriglyceridemia, and DM were more prevalent in HIV group without PIs. Time of disease and time of HAART were higher in HIV+ patients using PIs.

Common carotid IMT measurement is still the reference method for detection of atherosclerosis, since the femoral artery IMT showed a moderate sensitivity and low specificity to the former.
